# Assessing the effectiveness and cost effectiveness of subcutaneous nerve stimulation in patients with predominant back pain due to failed back surgery syndrome (SubQStim study): study protocol for a multicenter randomized controlled trial

**DOI:** 10.1186/1745-6215-14-189

**Published:** 2013-06-25

**Authors:** Sam Eldabe, Michael Kern, Wilco Peul, Colin Green, Kristi Winterfeldt, Rod S Taylor

**Affiliations:** 1 The James Cook Hospital, Middlesbrough, UK; 2 Elisabethinen Hospital, Graz, Austria; 3 Leiden University Medical Center and Medical Center, The Hague, The Netherlands; 4 Exeter Medical School, University of Exeter, Exeter, UK; 5 Medtronic, Inc, Tolochenaz, Switzerland; 6 Institute of Health Services Research, University of Exeter Medical School, Veysey Building, Salmon Pool Lane, Exeter EX2 4SG, UK

**Keywords:** Chronic Back Pain, Failed Back Surgery Syndrome, Peripheral Nerve Stimulation, Randomized Controlled Trial, Subcutaneous Stimulation

## Abstract

**Background:**

Chronic radicular pain can be effectively treated with spinal cord stimulation, but this therapy is not always sufficient for chronic back pain. Subcutaneous nerve stimulation (SQS) refers to the placement of percutaneous leads in the subcutaneous tissue within the area of pain. Case series data show that failed back surgery syndrome (FBSS) patients experience clinically important levels of pain relief following SQS and may also reduce their levels of analgesic therapy and experience functional well-being. However, to date, there is no randomized controlled trial evidence to support the use of SQS in FBSS.

**Methods/Design:**

The SubQStim study is a multicenter randomized controlled trial comparing SQS plus optimized medical management (‘SQS arm’) versus optimized medical management alone (‘OMM arm’) in patients with predominant back pain due to FBSS. Up to 400 patients will be recruited from approximately 33 centers in Europe and Australia and will be randomized 1:1 to the SQS or OMM arms. After 9 months, patients who fail to reach the primary outcome will be allowed to switch treatments. Patients will be evaluated at baseline (prior to randomization) and at 1, 3, 6, 9, 12, 18, 24, and 36 months after randomization. The primary outcome is the proportion of patients at 9 months with a ≥50% reduction in back pain intensity compared to baseline. The secondary outcomes are: back and leg pain intensity score, functional disability, health-related quality of life, patient satisfaction, patient global impression of change, healthcare resource utilization/costs, cost-effectiveness analysis and adverse events. Outcomes arms will be compared between SQS and OMM arms at all evaluation points up to and including 9 months. After the 9-month assessment visit, the main analytic focus will be to compare within patient changes in outcomes relative to baseline.

**Discussion:**

The SubQStim trial began patient recruitment in November 2012. Recruitment is expected to close in late 2014.

**Trial registration:**

ClinicalTrials.gov NCT01711619

## Background

Chronic back and leg pain (CBLP) is a diffusely defined group of pain conditions, ranging from chronic low back (‘axial’) pain to persisting hip, buttock and leg (‘radicular’) pain and often consists of a combination of both [1]. It is estimated that 10% to 40% of patients who have undergone lumbar spine surgery still experience CBLP: the so-called ‘failed back surgery syndrome’ (FBSS). CBLP and FBSS can be very difficult to manage for patients and clinicians and is associated with substantive negative impact on a patient’s health-related quality and well-being [2,3]. Furthermore, the management of CBLP places a high economic burden on both healthcare systems and society [4].

Greater success has been reported in the treatment of patients with CBLP and FBSS that present with radicular (or leg) pain rather than with axial (or low back) pain [5,6]. Randomized controlled trials (RCTs) have shown spinal cord stimulation to be a clinically effective adjunct to medical management and an alternative to a further operation in FBSS [7,8]. However, these studies have been limited to individuals who presented with predominant leg pain, excluding those with a chief issue of axial pain exceeding radicular pain. Other therapies have been applied to FBSS presenting as predominant back pain including peripheral neuromodulation.

Peripheral neuromodulation includes two distinct technical approaches: the lead can be placed percutaneously or via an open surgical technique. Peripheral nerve stimulation (PNS) utilizing an open surgical technique was first reported in 1967 [9]. This technique involves the use of a device to provide stimulation to a single peripheral nerve, with the goal of producing stimulation-induced paresthesia and relief of pain within the sensory distribution of that nerve [9,10]. Subcutaneous stimulation (SQS) (also known as ‘peripheral nerve field stimulation’ or ‘regional field stimulation’ or ‘target field stimulation’), a more recent advance in neuromodulation, targets not a single nerve, but rather the area of pain perception itself. In SQS, percutaneous leads are placed in the subcutaneous tissue within the area of pain perception. The mechanism of SQS-mediated paresthesia remains to be fully elucidated. However, it is thought that its effect arises from the creation of an electrical field that results in decreased nociceptive input which may impact on local blood flow, block cell membrane depolarization, and/or alter neurotransmitter release and reuptake, thus altering nociceptive information as it is transmitted to the central nervous system [11].

The remainder of this paper focuses on SQS.

### Systematic review

To the best of our knowledge, no previous systematic review of SQS for CBLP or FBSS has been published. We, therefore, undertook a comprehensive search of a number of electronic databases including MEDLINE, EMBASE and the Cochrane Library. Our search included a combination of Medical Subject Headings that included: ‘failed back surgery syndrome’, OR ‘back pain’ OR ‘chronic leg pain’ OR ‘post laminectomy’ AND ‘peripheral nerve stimulation’ OR ‘electric stimulation’ OR ‘subcutaneous stimulation’. Issues of ‘Neuromodulation’ were hand searched and the reference list checked from the Medtronic dossier of evidence of peripheral nerve stimulation that was submitted to the TuV in 2011 for purposes of obtaining Conformité Européenne (CE) Mark for percutaneous PNS [unpublished results].

The study selection process is shown in Additional file 1: Figure S1. Our searches identified 1,030 citations up to June 2012. Following exclusions (that is, non-English publications, hybrid SQS and spinal cord stimulation (SCS) systems, non-CBLP or FBSS indications, case reports and studies published only as abstracts) we identified eight studies (nine publications) that reported efficacy and device-related complication outcomes with SQS (see Table 1) [12-20]. Study selection, data extraction and quality assessment was undertaken by a single author and then checked for accuracy by a second.

**Table 1:** Systematic review: summary of characteristics of included studies

**Study**		**Population**			
**Lead author (year), reference, country, type and recruitment dates**	**Study design**	**Implanted N (tested N)**	**Description (as stated by authors) and indication**	**Age, gender, post back surgery**	**Baseline VAS/NRS**^**a**^
Burgher [17] USA, single center, August 2009 to December 2010	Retrospective case series	6 (10)	‘Axial back and neck pain’	Mean 53 years, 20% male, not reported	Not reported
			‘All patients had failed more conservative treatment…’		
			All predominantly axial pain		
Campbell [12] USA, single center, July 1974 to August 1975	Case series	10 (NR)	‘Low back pain syndrome with sciatica’	Mean 48 years, 20% male, not reported	Not reported
			‘Persistent disabling pain despite all traditional medical and surgery’		
Falco [16] USA, single center, August 2008 to April 2009	Case series	18^b^ (28)	‘Chronic [non-appendicular] pain that had not responded to conservative or surgical treatment and whose pain significantly impacted their quality of life’	Mean 56 years, 28% male, not reported	Mean 9.1
			‘Many of the patients had multiple sources and areas of chronic pain for years’		
			‘…in several cases, [failed] lumbar spine surgery.’		
Paicius [13] USA, single center. May 2005 to September 2006	Case series	6 (NR)	‘History of chronic low back pain’	Mean 63 years, 50% male, 83%	Mean 9.1
			Majority of patients followed lumbar surgery		
Sator-Katzenchlager [14] Austria, multicenter June 1999 to February 2007	Retrospective case series	66^c^ (NR)	‘Failed back surgery syndrome’ and ‘low back pain’	Mean 59 years, 48% male, 56%	Mean 8.2
			‘Prior failure of systemic or less invasive \treatments….and no indication for further surgery’		
Verrills [15,19] Australia, single center, dates not reported	Retrospective case series	23^b^ (NR)	‘Failure to respond to conservative treatment….almost all patients had also failed surgical procedures’	Mean 58 years, 39% male, not reported	Not reported
			‘Low back pain’		
Verrills [18,19] Australia, single center, dates not reported	Prospective case series	44^d^ (NR)	‘Lumbrosacral pain’	Range 27 to 88 years, 43% male, not reported	Mean 8.1
			‘Failure to respond to other conservative therapy’		
Yakovlev [20] USA, single center August 2007 to July 2009	Retrospective case series	18 (NR)	‘Chronic low back and lower extremity pain associated with PLS after multilevel spinal surgical procedures’	Mean 62 years, 61% male, 100%	12

Population indicates number of patients with CLBP pain unless other stated.

^a^0 to 10 scale.

^b^Mixed indication case series: unclear N CLBP/FBSS.

^b^Mixed indication case series of N = 111.

^d^Mixed indication case series of N = 100.

*(C)LBP* (chronic) low back pain, *FBSS* failed back surgery syndrome, *NR* not reported, *NRS* numeric rating scale, *PLS* post laminectomy syndrome, *VAS* visual analog scale.

All included studies employed a case series design (that is, reported outcomes in a cohort of patients before and after SQS and no control group) and included a total of 191 patients with CBLP implanted with SQS, of which a proportion of patients experienced CBLP following lumbar surgery, that is, FBSS. Although the precise location of pain was often not clearly stated, studies appear to recruit patients specifically with a back component of pain. Only one study explicitly stated that they included individuals with both back and leg pain [20]. Studies assessed outcomes before and after SQS implantation, with follow-up ranging from 3 to 12 months.

The quality of included studies was assessed on the basis of the following five criteria: (1) prospective design, (2) consecutive or random sampling, (3) explicit statement of inclusion/exclusion criteria, (4) independent outcome assessment and (5) clear statement of loss to follow-up/withdrawals [21]. Details of study methodology were generally poorly reported and limited our ability to assess study quality. A table summarizing quality assessment is provided as Additional file 1: Table S1.

Studies consistently reported that patients experienced pain relief following SQS implantation (see Table 2). Across the 6 studies reporting pain visual analog scale (VAS) values, the average baseline score varied from 6.8 to 9.1 (on a 10-point scale) [13-16,18,20]. Following SQS, the reduction in mean VAS ranged from 4.2 to 1.7. Five studies also reported improvements in the level of analgesia medication, [14,17-20] and three reported improvements in functional capacity compared to before SQS [13,16,18].

**Table 2:** Systematic review: summary of efficacy results

**Lead author, year and reference**	**Follow-up**	**Pain outcome**			**Other outcomes**
		**Pre-VAS/NRS, mean (SD)**	**Post-VAS/NRS, mean (SD), *P *value**^**a**^	**Percentage pain relief**	
Burgher [17]	Mean 4.5 months	Not reported	Not reported	Mean 45% (range: 20 to 80%) (n = 10)	All patients reported to be ‘somewhat’ or ‘very satisfied’ with outcome; 1/6 patients reported reduction in opiate intake
Campbell [12]	Mean 12.8 months	Not reported	Not reported	Not reported	0/10 achieved ‘excellent’ outcome on pain relief; 2/10 achieved ‘partial success’ of pain relief; 8/10 ‘failure’ of pain relief
Falco [16]	Mean 3 months	9.1 (1.1) (n = 18)	1.2 (1.0) (n = 18), <0.0001^a^	Not reported	Pain medication usage dropped in 18/18 patients with 7/18 taking no pain medication; ODI: before treatment, mean 33.6 (SD 6.4) and after treatment, mean 19.7 (9.0)
Paicius [13]	Not reported	6.8 (2.3) (n = 6)	2.5 (0.6) (n = 4), 0.07^ab^	Not reported	‘All patients experienced a significant….increase in their ability to function’
Sator-Katzenchlager [14]	3 months	FBSS 8.0 (1.4) (n = 37); LBP 8.3 (0.9) (n = 29)	FBSS 3.3 (2.1), <0.0001; LBP 4.2 (2.2) (n = 29), <0.0001	Not reported	Significant reduction in WHO analgesic medication score
Verrills [15,19]	Mean 7.6 months	7.5 (1.2) (n = 28)	3.5 (2.0) (n = 28), <0.05	Mean 60% 16/23 (70%) ≥50% pain relief	2/23 (7%) increased capacity to perform work; 16/23 (70%) reduced analgesia; 20/23 (87%) ‘satisfied’ or better
Verrills [18,19]	Mean 7.2 months	7.0 (1.3) (n = 44)	3.7 (2.6) (n = 44), <0.0001^ab^	Not reported	Significant improvement (*P* <0.03) in disability as assessed by ODI
Yakovlev [20]	1 and 12 months	7.4 (1.0) (n = 18)	1 month 2.6 (0.8) (n = 18), <0.0001^ab^; 12 months 1.7 (0.6) (n = 18), <0.0001^ab^	18/18 (100%) ≥50% pain relief at 1 and 12 months	Decreased or discontinued use of pain medication 16/18 (89%)

^a^*P* value for pre/post treatment comparison.

^b^Paired t test undertaken by authors of the present report.

*FBSS* failed back surgery syndrome, *LBP* low back pain, *NRS* numeric rating scale, *ODI* Oswestry Disability Index, *VAS* visual analog scale, *WHO* World Health Organization.

Complications were inconsistently reported across studies. Across the 3 studies reporting data, a total of 41 out of 224 (18%) patients experienced 1 or more device-related complication [14,18,19]. Risks of specific complications were as follows: lead migration, 19/245 (8%, 5 studies [14,16-19]); other lead complications (for example, fracture), 10/211 (5%, 2 studies [14,19]); hardware erosion, 0/13 (0%, 1 study [10]); infection, 9/258 (3.5%, 6 studies [12,14,17-20]); and device migration 1/113 (<1%, 2 studies [12,19]).

In summary, our systematic review identified a growing body of international prospective and retrospective case series data showing that CBLP and FBSS patients experience pain relief following SQS, a reduction in their analgesic therapy and an improvement in functional well-being over the short to medium term. The reduction in pain observed across studies compares favorably with the clinically important change of two or more units (on a scale of 0 to 10) cited by the IMMPACT (‘Initiative on Methods, Measurement, and Pain Assessment in Clinical Trials’) guidelines on outcome measures for pain trials [21]. Furthermore, SQS appears to be associated with a level of device-related complications similar, or lower, to that seen with spinal cord stimulation [22,23]. However, given the current absence of level I clinical and economic evidence for SQS, this overview supports the urgent need for a well-conducted RCT and parallel economic evaluation.

### Aims and objectives

The SubQStim study is a multicenter RCT comparing the clinical effectiveness of peripheral nerve stimulation utilizing a subcutaneous lead implant technique plus optimized medical management (‘SQS arm’) versus optimal medical management alone (‘OMM arm’). Patients with back pain due to FBSS will be randomized to the SQS or OMM arms. Patients that fail to achieve the primary outcome (that is, ≥50% reduction in back pain intensity) at 9 months post randomization will be allowed to switch treatments. All patients will be followed up for 36 months post randomization.

The primary objective of SubQStim study is to compare the proportion of patients achieving the primary outcome at 9 months post randomization in SQS and OMM arms. Secondary objectives are to: (1) compare the primary outcome between SQS and OMM arms at 3 months and 6 months post randomization; (2) compare secondary outcomes (functional disability, health-related quality of life, patient satisfaction, patient global impression of change, healthcare utilization/costs, use of pain mediation and non-invasive pain treatment) between SQS and OMM arms at 3, 6 and 9 months post randomization; (3) assess the value of trial stimulation in predicting 9-month post-randomization primary outcome response in the SQS arm; (4) assess the trend in device programming parameters up to 9 months post randomization; (5) assess within-patient changes (that is, compared to baseline/prior to randomization) in primary and secondary outcomes from 9 to 36 months post randomization; (6) quantify the level of SQS-related and non-SQS related adverse events at all follow-up timings up to 36 months post randomization.

## Methods/Design

### Design and setting

**Figure 1:**
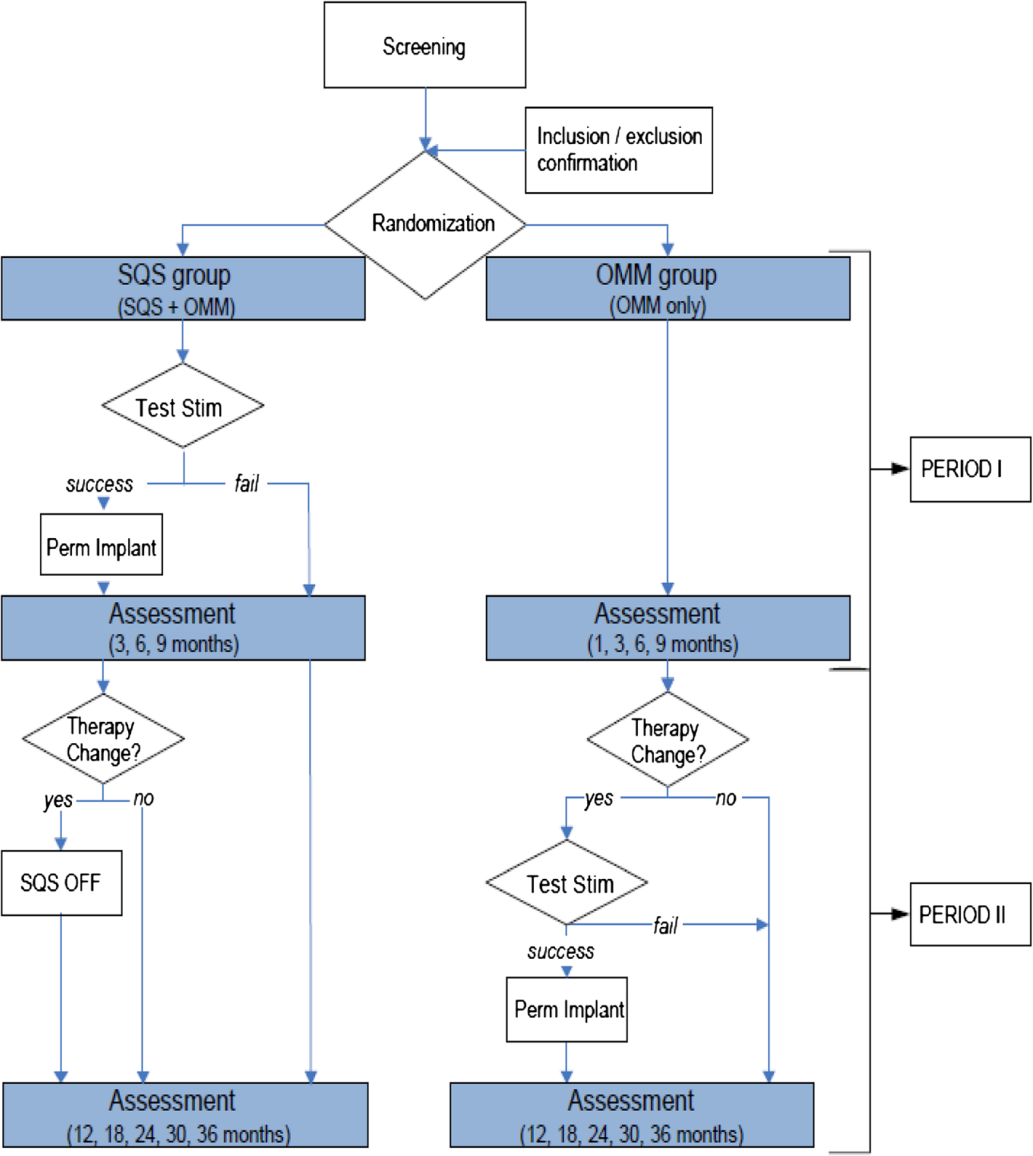
Study design.

Patients will be randomized 1:1 to peripheral nerve stimulation utilizing a subcutaneous lead implant technique plus optimized medical management (‘SQS arm’) or optimized medical management alone (‘OMM arm’) and followed for 9 months (period I: randomized comparative period). At 9 months after randomization, if patients in either arm fail to meet certain criteria (see below) they may switch treatment; that is, those in the OMM arm receive SQS or those in the SQS arm have their device turned off and/or explanted. Patients will be followed up until 36 months post randomization (period II: long-term follow-up period). The study design is summarized in Figure 1.

The SubQStim study will be conducted in specialized investigational centers. The principal investigator in each site will be experienced in implanting spinal cord stimulation systems and/or SQS systems or specializes in the diagnosis and ongoing treatment of back pain (for example, pain management physician, anesthesiologist, neurosurgeon, and orthopedic surgeon). It is anticipated that approximately 33 centers (30 European site and 3 Australian sites) will participate. No single center can exceed 15% of the total evaluable patients (that is, patients with assessment of the primary outcome).

### Selection of patients

The target population is patients experiencing intractable chronic back pain due to FBSS.

At the screening visit, patients will be assessed to determine if they meet all of the inclusion criteria and are, therefore, eligible to participate in the study. Potential patients who meet any of the exclusion criteria will not be eligible to participate in the study.

#### *Inclusion criteria*

Inclusion criteria are: signed and dated the Patient Informed Consent/Patient Information Sheet prior to any study-related activities being conducted; ≥18 years of age at time of informed consent; minimum average back pain intensity of ≥50 mm assessed by VAS at screening visit; willing and available to attend visits as scheduled and to comply with the study protocol; willing and able to undergo assessments as part of the evaluation for eligibility and endpoints; willing and able to use the external neurostimulator, recharging equipment (if applicable), and patient programmer per the schedule required by the protocol; diagnosed as having FBSS, that is, has had persistent pain for 6 months following most recent anatomically correct, successful surgery and there are no further therapeutic surgical options available as assessed by appropriate investigation; based on the opinion of the Principal Investigator, patient’s back pain is considered intractable and has been adequately treated, as defined by: failed an appropriate trial of at least three different classes of back pain treatments (pharmaceutical and/or non-pharmaceutical); based on the opinion of the implanting physician, an appropriate implant candidate for the SQS system.

#### *Exclusion criteria*

Exclusion criteria are: has been or is being treated with spinal cord stimulation, subcutaneous nerve stimulation, peripheral nerve stimulation or an implantable intrathecal drug delivery system; leg pain score >30 mm assessed by VAS at screening visit; evidence of an active disruptive psychiatric disorder that is significant enough to impact the perception of pain, compliance to intervention and/or ability to evaluate treatment outcome, as determined by the investigator; pain condition unrelated to FBSS that is severe enough to overshadow the FBSS pain in the opinion of the investigator; evidence of a spinal instability or anatomic compression that requires further surgery; spinal fusion at more than three vertebral levels; currently enrolled in or plans to enroll in any concurrent drug and/or device study that may confound the results of this study; allergic or has shown hypersensitivity to any materials of the device system which come in contact with the body; history of coagulation disorder or lupus erythematosus; involved in current litigation regarding back pain.

Patients meeting the inclusion/exclusion criteria will complete a baseline diary questionnaire for 7 days. During the baseline diary, the subject must: (1) report an average back pain score ≥50 mm, (2) report an average leg pain score ≤30 mm, and (3) complete the diary for at least 5 days.

### Interventions

Patients randomized to both arms will have their medical management optimized during the 9-month treatment period, beginning at randomization. Optimized medical management for each subject will be defined by the investigator. Optimized medical management does not include back reoperation or the implantation of medical devices (for example, neurostimulation or intrathecal drug delivery). For example, this may include physiotherapy, transcutaneous electrical nerve stimulation, pharmaceutical treatments or acupuncture.

Patients randomized to the SQS arm will undergo a test stimulation implant and an at-home trialing period. During the trialing period, patients will be programmed according to their optimal programming parameters and will be able to adjust their stimulation via the patient programmer, within the settings programmed by the physician. Prior to the permanent implant, all implanted patients will discuss the trialing period with the principal physician and complete a VAS for both back and leg pain. A stimulation trial is only considered successful if the following occurs: the patient agrees to proceed with the permanent implant and finds the feeling of paresthesia comfortable and has a pain reduction of ≥30% as measured by VAS or some reduction in pain as measured by VAS, along with improved function, and/or improved health-related quality of life, and/or a reduction in pain medications as assessed by the principal investigator. Given the lack of evidence of the predictive value of a stimulation trial [24], we choose a trial cut off of 30% (rather than the conventional 50%) to enable a larger percentage of patients to progress to permanent implant. All SQS arm subjects who had successful trial stimulation, will be implanted with a neurostimulator (Medtronic, Inc., Minneapolis, MN, USA).. The final system implanted will consist of a Medtronic pulse generator (rechargeable or primary cell) attached either directly or via extension cable to two subcutaneous leads (quadripolar or octopolar). Patients should receive their permanent implant within 10 weeks of randomization. In the event that a patient does not proceed with the permanent implant, the patient will stay in the study and will be followed according the scheduled visits, unless the patient withdraws consent.

The following SQS-related equipment will be used in this study: The RestoreAdvanced®, PrimeAdvanced®, RestoreUltra®, and RestoreSensor® neurostimulators, 1 × 8 Standard and Quad Plus® leads (all Medtronic, Inc., Minneapolis, MN, USA)., and all current commercially available internal and external accessories approved for use for Peripheral Nerve Stimulation, and all future commercially available Medtronic neurostimulation devices, leads and accessories approved for use for Peripheral Nerve Stimulation.

### Treatment switch criteria

The investigator criteria to inform patient treatment switch after 9 months post randomization or later are as follows: if <30% pain relief on VAS: switchover is normally allowed without any further justification; if 30% to 50% pain relief on VAS: switchover is normally allowed if the patient has another reason for not responding to randomized arm, such as: no perceived improvement in quality of life, disability or medication use and/or side effects from treatments; if >50% pain relief on VAS: switchover is normally not allowed.

### Assessments

Patients will be assessed prior to randomization (baseline) and at follow-up visits at 3, 6, 9, 12, 18, 24, 30 and 36 months post randomization. Assessments will require that patients visit the local investigational center and have their data collected via an electronic case report form. Table 3 provides a summary of the data collection process and timing.

**Table 3:** SubQStim study: summary of data collection

**Study requirements**	**Screening**	**Baseline**	**Test stimulation implant**	**Permanent implant**	**Wound check**	**1 month**	**3 months**	**6 months**	**9 months**	**12 months**	**18 and 24 months**	**30 and 36 months**	**Unscheduled**	**Discontinued**
Patient informed consent/patient information sheet	X													
Screening inclusion/exclusion criteria	X													
Demographics	X													
Physical examination	X													
Medical history	X													
HCU history	X													
VAS (point in time measurement only)	X												X	X
Back pain map	X		X^a^	X^a^	X	X	X	X	X	X	X	X	X	X
Paresthesia map (implanted subjects only)			X	X^a^	X	X	X	X	X	X	X	X	X	X
Confirmation of 7-day baseline dairy inclusion criteria		X												
Randomization assignment (after questionnaires)		X												
HADS		X												
painDETECT		X												
EQ-5D		X					X	X	X	X	X	X	X	X
ODI		X					X	X	X	X	X	X		
SF-36		X					X	X	X	X	X	X		
PGIC		X					X	X	X	X	X	X	X	X
Implant information			X^a^	X^a^										
Postoperative radiography				X										
Subject satisfaction							X	X	X	X	X	X	X	X
7-day diary completion							X	X	X	X	X	X		
Initial and final interrogations and uploads			X	X	X	X	X	X	X	X	X	X	X	X
Medication assessment		X	X	X	X	X	X	X	X	X	X	X	X	X
Protocol deviations		X	X	X	X	X	X	X	X	X	X	X	X	X
Event assessment (adverse events)		X	X	X	X	X	X	X	X	X	X	X	X	X
HCU		X	X	X	X	X	X	X	X	X	X	X	X	X

*EQ-5D* EuroQoL five dimensions, *HADS* Hospital Anxiety and Depression Scale, *HCU* healthcare utilization, *ODI* Oswestry Disability Index, *PGIC* Patient Global Impression of Change, *SF-36* Short-Form 36, *VAS* visual analog scale. ^a^Pre-implant and post-implant as indicated; otherwise, post-implant or post-reprogramming only.

#### *Primary outcome*

The proportion of subjects with a ≥50% reduction in back pain intensity (VAS) from baseline to the 9 months post randomization. Patients will complete a pain diary, comprising a VAS scale, three times daily during the 7 days prior to their visit. The average VAS during that period will be calculated as the ratio between the sum of recorded VAS and the number of VAS measured. The percentage pain reduction will be calculated as 100*(VAS_0_-VAS_V_)/VAS_0_, where VAS_0_ is the average VAS at baseline visit and VAS_v_ is the average VAS at subsequent visits.

#### *Secondary outcomes*

Secondary outcomes are: pain relief: ≥50% back pain relief at all visits in addition to 9 months, ≥30% back pain relief at all time points; pain intensity score: back and leg VAS score via patient diary at all time points; functional disability: Oswestry Disability Index (ODI) version 2 [24] at all time points; health-related quality of life: Short-Form 36 (SF-36) [25] and EQ-5D-5 L [26] at all time points; patient satisfaction (‘are you satisfied with the pain relief provided by your treatment?’ and ‘based on your experience so far, would you have agreed to this treatment?’) [7] at all follow-up time points; Patient Global Impression of Change (PGIC) [27] at all follow-up time points; healthcare utilization (HCU), that is, concomitant drug treatment for pain relief (opioid, non-steroidal anti-inflammatory drugs (NSAIDs), antidepressant drugs, anticonvulsants), need for physical therapy or other non-drug treatments) [28] at all time points; adverse events: both device-related and non-device-related adverse events and complications will be collected. The nature and frequency of events will be documented and an Adverse Events Committee will adjudicate all events.

#### *Process measures*

At the screening and baseline visits, the following additional information will be collected: patient demographics (for example, age, gender, duration of back pain), neuropathic pain severity (Douleur Neuropathique en 4 Questions (DN4)), [29] mood (Hospital Anxiety and Depression Scale (HADS)) [30] and level of previous healthcare utilization. Patients who proceed to device implantation will have the following information collected: back pain and paresthesia maps, radiographic images, parameter settings, lead location, and device implant information (that is, model, serial number). At subsequent visits, clinical and patient programmer data will be downloaded. All medical and alternative treatments received as optimized medical management will be recorded at each follow-up visit.

Unscheduled patient visits could occur between scheduled study follow-up visits for the following reasons: patient discontinuation from the study, device reprogramming and management of device-related complications, or revisions of non-device treatment.

### Sample size and power calculations

A 20% difference in the primary outcome between the SQS and OMM arms is considered relevant. Assuming that 40% of the patients receiving OMM alone achieve the primary outcome, the study needs to recruit a total of 350 patients to detect a clinically important difference at the 5% alpha level and 90% power allowing for 20% attrition. However, it is expected that the difference between the two arms of the trial may be larger, that is, 25% or 30%. Two interim analyses are, therefore, planned at 140 and 220 evaluable patients (that is, with completed primary outcome) to provide 80% power in order to detect a 30% difference, and 85% power to detect a 25% difference (respectively), at 2-sided 1% alpha level. In order to ensure an overall alpha of two-sided 5%, the final analysis will be performed at two-sided 3% alpha [31]. In order to undertake 2 interim analyses and a final analysis, a total of 400 patients (N = 200 SQS arm and N = 200 OMM arm) will be required. These interim and final analyses are summarized in Table 4.

**Table 4:** SubQStim study: sample size and power calculations

**Analysis**	**Expected difference between groups**	**Power (1-β)**	**Nominal error risk (α)**	**Proportion of responders**		**Sample size**	
				**OMM arm**	**SQS arm**	**Evaluable patients**^**a**^	**Enrolled (20% attrition rate)**
Interim 1	30%	80%	1%	35%	65%	140	176
Interim 2	25%	85%	1%	37.5%	62.5%	220	276
Final	20%	90%	3%	40%	60%	314	392
For reference: one-shot analysis	20%	90%	5%	40%	60%	280	350

^a^Patients with completed primary outcome data.

### Procedures to minimize bias

Computer generated random allocation schedules will be preprepared for each investigational center. The randomization schedule will be centrally held and allocation concealment will be maintained by an online database system that automatically assigns the patient to one of the two arms on confirmation by research nurse at the end of the baseline visit. The randomization schedules will be stratified to maintain a 1:1 balance between the treatment and control arm within each center and in the study as a whole. Patients will be randomized sequentially based upon randomization date and time.

Given the nature of both the intervention and control treatments, it is unfortunately not possible to blind either patients, or clinicians.

If a patient misses a study visit, the center’s study staff will make three attempts (one in writing and sent via a traceable method) to bring them in for a study visit. The numbers and reasons for dropouts and losses to follow-up will be reported by each arm of the study.

All study deviations (that is, an event where the investigator, or center personnel, did not conduct the study according to the Clinical Investigational Plan, Protocol, or Clinical Investigation Agreement) will be recorded and the reasons documented.

### Data analysis

#### *Statistical analysis*

The intent-to-treat patient set (ITT) consists of all patients as they were randomized into the study. The per-treatment patient set (PT) consists of all patients of the ITT patient set for whom no major protocol deviation was reported. Patients who underwent a treatment switch, that is, who had initially been randomized to the OMM arm and switched to the SQS arm, or *vice versa*, will be included in this patient set. They will be analyzed according to treatment actually received.

The primary effectiveness analysis will be based on a between group comparison of primary and secondary outcomes up to, and including, the 9-month visit using the ITT patient data set [32]. For continuous outcomes, analytic models will be adjusted for baseline outcome score.

Sensitivity analyses will be undertaken using the same analytic models used in the primary analysis and based on the PT data set. In case of a discrepancy between the two analyses, a closer analysis of the data, of those patients from which the discrepancies result, will be performed. An additional sensitivity ‘modified ITT’ analysis will be performed for the primary outcome, considering patients randomized to the SQS arm but not definitively implanted as ‘failures’.

In phase II of the study (beyond 9-month visit) the analytic focus will be a within-person approach, that is, comparison of primary and secondary outcomes at follow-up, relative to baseline. In addition, the percentage of subjects who switched treatments after the 9-month follow-up visit, and the time to switchover, will be compared between OMM and SQS arms.

The study will seek to minimize missing data by conducting completeness checks on study data and patient questionnaires, with follow-up on any missing data. It is not possible to predict which will be the most effective method, therefore, we will undertake sensitivity analyses to examine different methods of missing data imputation, such as mean of nearby points (moving average), last value carried forward, doubly robust estimators or the best or the worst values that are not in favor of the SQS arm [33,34].

The ‘safety patient set’ consists of all patients of the ITT dataset, who started any of the study procedures independent of the treatment they had been randomized to. Complications and adverse events will be reported descriptively.

All statistical tests will be interpreted at the two-sided α = 5% level and results reported as means and 95% confidence intervals. A more detailed exposition of study statistical analyses will be presented in a Statistical Analysis Plan to be completed prior to any data analysis.

#### *Economic evaluation*

Economic analysis alongside the RCT will estimate the cost effectiveness of SQS plus OMM versus OMM alone. The primary economic endpoints will be the cost per quality-adjusted life-years (QALYs) gained, and cost per additional unit of change in the primary outcome (that is, per unit change in pain VAS and change in proportion with ≥50% reduction in back pain intensity). Primary economic analyses will be at 9-month follow-up, with secondary analyses comparing costs over longer-term follow-up. Cost-effectiveness analyses will be undertaken using the perspective of a third party payer, for example, health service provider/payer perspective.

Within-trial data collection will inform estimates of intervention related resource, and resource use data will be combined with appropriate unit costs to estimate SQS intervention costs, in addition to OMM. Estimates of costs associated with other resource use will be informed by the HCU data collected within trial. Participant self-report data on EQ-5D-5 L [25] and SF-36 [26] will be combined with country specific tariffs to estimate health state values, and mean (and standard deviation) health state value for each arm at assessment point. The EQ-5D-5 L will be used as the primary outcome measure for health state values, with SF-36 (SF-6D, a utility score calculated from SF-36) used in sensitivity analyses. Health state values will be used to estimate QALY data over appropriate time periods. QALY data will be estimated using the area under the curve approach [35]. Comparison of costs, mean health state value, and QALY, over time will be adjusted for baseline value consistent with the statistical analysis plan.

Where analyses are conducted over a 12 month or longer time horizon, future costs and outcomes will be discounted at rates appropriate for country level analyses. Analyses of costs and outcomes will consider uncertainty in parameter estimates, assumptions (for example, device lifetime), and limitations with the data, using extensive sensitivity analyses. The net benefit statistic will be used to present cost-effectiveness acceptability curves and confidence intervals on cost effectiveness against a range of estimates of willingness to pay per additional unit of outcome (for example, cost per QALY threshold values) [36,37].

### Ethics and governance

Prior to enrolling subjects in this study, each investigation center’s Medical Ethical Committee will be required to approve the current study Clinical Investigation Plan and the Patient Information and Informed Consent Form. Any other written information to be provided to the subjects and, if applicable, any materials used to recruit subjects will also require ethical approval. Furthermore, such approval must be received in the form of a letter and provided to the study Sponsor (Medtronic Inc.) before commencement of the study at an investigation center.

At each center, investigators must obtain written informed consent prior to subjecting patients to any study related activity.

An independent Adverse Event Committee will review all adverse events. This Committee will consist of a minimum of three physicians independent of the Sponsor. The responsibilities of the Adverse Event Committee include, but are not limited to, the following: review all reported adverse events and their classification on a periodic basis; upon request, advise the Sponsor about the potential clinical impact of an observed, unintended device performance and cases of serious adverse device events.

Finally, clinical monitors, as a representative of the Sponsor, will visit the principal investigators and/or subinvestigators and study centers at periodic intervals to ensure compliance to the investigational plan and study agreements, adherence to current International Organization for Standardization, Declaration of Helsinki regulations and standard operational procedures, and check accuracy of study data.

## Discussion

RCTs have provided evidence to indicate spinal cord stimulation to be a clinically effective adjunct to medical management, and an alternative to a further operation for patients experiencing FBSS [7,8]. Given this level I evidence, payers such as the National Institute for Health and Clinical Excellence (NICE) in the UK have recommended SCS as both a clinically and cost-effective therapy for the management of FBSS [38-40].

Nevertheless, while chronic leg (or radicular) pain can be effectively treated with SCS, this therapy is not always sufficient for chronic back pain. The existing trials of spinal cord stimulation in patients with FBSS, recruited individuals who presented with predominant leg pain and excluded those with predominant back pain [7,8].

Our systematic review identified 9 cases series studies of SQS in a total of 191 patients with chronic back pain. Importantly, these studies show that SQS implantation can result in clinically significant levels of pain relief in predominant back pain that is due to FBSS. The SubQStim study is the first RCT to assess the effectiveness and cost effectiveness of SQS in this patient population.

## Trial status

The SubQStim trial began patient recruitment in November 2012. Recruitment is expected to close in late 2014. It is anticipated that primary endpoint findings will be available in approximately 2016.

## Abbreviations

CBLP: Chronic back and leg pain; CE: Conformité Européenne (European Conformity); DN4: Douleur Neuropathique en 4 Questions; FBSS: Failed back surgery syndrome; HADS: Hospital Anxiety and Depression Scale; HCU: Healthcare utilization; ITT: Intention to treat; LBP: Low back pain; MR: Magnetic resonance; ODI: Oswestry Disability Index; OMM: Optimal medical management; NSAIDs: Non-steroidal anti-inflammatory drugs; PGIC: Patient Global Impression of Change; PT: Per treatment; RCT: Randomized controlled trial; SF-36: Short-Form 36; SQS: Subcutaneous nerve stimulation; VAS: Visual analog scale; WHO: World Health Organization.

## Competing interests

SE, CG, MK, WP and RST have received consultancy fees from Medtronic. KW is a full-time employee of Medtronic.

## Authors’ contributions

SE is the overall study principal investigator. All authors are member of the SubQStim Trial Steering Committee and have participated in the study conception and design. All authors contributed to the writing of the study protocol and the drafting and editing of this manuscript. All authors read and approved the final manuscript.

## Supplementary Material

Additional file 1 **Figure S1.** Systematic review: summary of study selection. **Table S1**. Systematic review: quality assessment. Click here for file
