# Supplementary material for: Assessing the effectiveness and cost effectiveness of subcutaneous nerve stimulation in patients with predominant back pain due to failed back surgery syndrome (SubQStim study): study protocol for a multicenter randomized controlled trial
Source: Trials. 2013 Jun 25;14:189. doi: 10.1186/1745-6215-14-189 (PMC3700888; doi:10.1186/1745-6215-14-189)
Supplement: Additional file 1 — Figure S1. Systematic review: summary of study selection. Table S1. Systematic review: quality assessment. [file 1745-6215-14-189-S1.docx]

**E-resource**

**eFigure. Systematic review: summary of study selection**

Electronic & citation searches

Titles & abstracts N=1030

Excluded

N=984

MDT Review

Full papers reviewed

N=27*

Full papers reviewed

N=46

Excluded N=41

Non-English language N=1

Case report N=3

Indication N=1

Review N=7

Hybrid SCS N=8

TENs or PNS or Percut N=18

Other N=4

Excluded N=24

Case report N=7

Indication N=16

PNS N=1

Final included N=9^+^ papers)

Case series N=8 studies

Included

N=5 studies (N=5 papers)

Included

N=3 studies (N=4^+^ papers)

*Unique references not identified in electronic searches

^+^One paper containing data contained in other 2 studies and papers

**eTable. Systematic review: quality assessment**

| Study | **Prospective or retrospective** | **Consecutive or random sample?** | **Explicit statement of inclusion/exclusion criteria?*** | **Outcome assessment independent?** | **Loss to follow up/withdrawals (%)** |
| --- | --- | --- | --- | --- | --- |
| **Burgher (2012)^18^** | Retrospective | Consecutive | Yes | Not reported | 0 |
| **Campbell (1976)^13^** | Not reported | Not reported | No | Not reported | 0 |
| **Falco (2009)^17^** | Not reported | Consecutive | No | Not reported | 0 |
| **Paicius (2007)^14^** | Not reported | Not reported | No | Not reported | 0 |
| **Sator-Katzenchlager (2010)^15^** | Retrospective | Not reported | Yes | Not reported | 0 |
| **Verrills (2009a)^16,20^** | Retrospective | Consecutive | Yes | Not reported | 11 |
| **Verrills (2011)^19,20^** | Prospective | Consecutive | Yes | Not reported | 0 |
| **Yakovlev (2011)^21^** | Retrospective | Consecutive | No | Not reported | 0 |

*i.e. statement of duration, location and severity of pain

**Table 3. SubQStim study – summary of data collection**

| **Study Requirements** | **Screening** | **Baseline** | **Test Stim Implant** | **Permanent Implant** | **Wound Check** | **1 mo** | **3 mo** | **6 mo** | **9 mo** | **12 mo** | **18 and 24 mo** | **30 and 36 mo** | **Unscheduled** | **Discontinue** |
| --- | --- | --- | --- | --- | --- | --- | --- | --- | --- | --- | --- | --- | --- | --- |
| Patient Informed Consent / Patient Information Sheet | **X** |  |  |  |  |  |  |  |  |  |  |  |  |  |
| Screening Inclusion/Exclusion Criteria | **X** |  |  |  |  |  |  |  |  |  |  |  |  |  |
| Demographics | **X** |  |  |  |  |  |  |  |  |  |  |  |  |  |
| Physical examination | **X** |  |  |  |  |  |  |  |  |  |  |  |  |  |
| Medical history | **X** |  |  |  |  |  |  |  |  |  |  |  |  |  |
| HCU history | **X** |  |  |  |  |  |  |  |  |  |  |  |  |  |
| VAS (Point in time measurement only) | **X** |  |  |  |  |  |  |  |  |  |  |  | **X** | **X** |
| Back pain Map | **X** |  | **X^** | **X^** | **X** | **X** | **X** | **X** | **X** | **X** | **X** | **X** | **X** | **X** |
| Paresthesia map (implanted subjects only) |  |  | **X** | **X^** | **X** | **X** | **X** | **X** | **X** | **X** | **X** | **X** | **X** | **X** |
| Confirmation of 7 day baseline dairy inclusion criteria |  | **X** |  |  |  |  |  |  |  |  |  |  |  |  |
| Randomization assignment (after questionnaires) |  | **X** |  |  |  |  |  |  |  |  |  |  |  |  |
| HADS |  | **X** |  |  |  |  |  |  |  |  |  |  |  |  |
| painDETECT |  | **X** |  |  |  |  |  |  |  |  |  |  |  |  |
| EQ-5D |  | **X** |  |  |  |  | **X** | **X** | **X** | **X** | **X** | **X** | **X** | **X** |
| ODI |  | **X** |  |  |  |  | **X** | **X** | **X** | **X** | **X** | **X** |  |  |
| SF-36 |  | **X** |  |  |  |  | **X** | **X** | **X** | **X** | **X** | **X** |  |  |
| PGIC |  | **X** |  |  |  |  | **X** | **X** | **X** | **X** | **X** | **X** | **X** | **X** |
| Implant information |  |  | **X^^^** | **X^^^** |  |  |  |  |  |  |  |  |  |  |
| Post-operative radiography |  |  |  | **X** |  |  |  |  |  |  |  |  |  |  |
| Subject Satisfaction |  |  |  |  |  |  | **X** | **X** | **X** | **X** | **X** | **X** | **X** | **X** |
| 7-day diary completion |  |  |  |  |  |  | **X** | **X** | **X** | **X** | **X** | **X** |  |  |
| Initial & final interrogations and uploads |  |  | **X** | **X** | **X** | **X** | **X** | **X** | **X** | **X** | **X** | **X** | **X** | **X** |
| Medication assessment |  | **X** | **X** | **X** | **X** | **X** | **X** | **X** | **X** | **X** | **X** | **X** | **X** | **X** |
| Protocol deviations |  | **X** | **X** | **X** | **X** | **X** | **X** | **X** | **X** | **X** | **X** | **X** | **X** | **X** |
| Event assessment (adverse events) |  | **X** | **X** | **X** | **X** | **X** | **X** | **X** | **X** | **X** | **X** | **X** | **X** | **X** |
| HCU |  | **X** | **X** | **X** | **X** | **X** | **X** | **X** | **X** | **X** | **X** | **X** | **X** | **X** |

HCU: health care utilization; VAS: visual analogue scale; HADS: Hospital Anxiety and Depression Scale; ODI: Oswestry Disability Index; Patient Global Impression of Change; SF-36: Short-Form 36

**Table 4. SubQStim study - sample size and power calculations**

| **Analysis** | **Expected difference between groups** | **Power (1-β)** | **Nominal error risk (α)** | **Proportion of responders** | | **Sample size** | |
| --- | --- | --- | --- | --- | --- | --- | --- |
|  |  |  |  | **OMM arm** | **SQS arm** | **Evaluable patients** | **Enrolled (20% attrition rate)** |
| **Interim 1** | 30% | 80% | 1% | 35% | 65% | 140 | 176 |
| **Interim 2** | 25% | 85% | 1% | 37.5% | 62.5% | 220 | 276 |
| **Final** | 20% | 90% | 3% | 40% | 60% | 314 | 392 |
| *For reference: 1-shot analysis* | *20%* | *90%* | *5%* | *40%* | *60%* | *280* | *350* |
